# HEAL Africa score to predict failure of surgical repair of obstetric urethro-vaginal fistula in the Democratic Republic of the Congo

**DOI:** 10.1186/s12905-024-02948-w

**Published:** 2024-02-10

**Authors:** Justin Lussy Paluku, Susan A. Bartels, Jonathan ML. Kasereka, Barthelemy Kasi Aksanti, Eugénie Mukekulu Kamabu, Olivier Mukuku, Zacharie Kibendelwa Tsongo, Stanis Okitotsho Wembonyama, Charles Wembonyama Mpoy, Jeannot Sihalikyolo Juakali

**Affiliations:** 1 Department of Obstetrics and Gynecology, Faculty of Medicine, University of Goma, Goma, Democratic Republic of the Congo; 2 Department of Obstetrics and Gynecology, HEAL Africa Hospital, Goma, Democratic Republic of the Congo; 3 Departments of Emergency Medicine and Public Health Sciences, Queen’s University, Kingston, Canada; 4 Department of Orthopedics and Trauma, HEAL Africa Hospital, Goma, Democratic Republic of the Congo; 5 Institut Supérieur des Techniques Médicales, Lubumbashi, Democratic Republic of the Congo; 6 Department of Internal Medicine, HEAL Africa Hospital, Goma, Democratic Republic of the Congo; 7 Department of Internal Medicine, Faculty of Medicine, University of Kisangani, Kisangani, Democratic Republic of the Congo; 8 Department of Pediatrics, Faculty of Medicine, University of Lubumbashi, Lubumbashi, Democratic Republic of the Congo; 9 Department of Public Health, Faculty of Medicine, University of Lubumbashi, Lubumbashi, Democratic Republic of the Congo; 10 Department of Obstetrics and Gynecology, Faculty of Medicine, University of Lubumbashi, Lubumbashi, Democratic Republic of the Congo; 11 Department of Obstetrics and Gynecology, Faculty of Medicine, University of Kisangani, Kisangani, Democratic Republic of the Congo

**Keywords:** HEAL Africa score, Obstetric urethro-vaginal fistula, Failure of surgical repair, DRC

## Abstract

**Introduction:**

Obstetric fistula (OF) repair surgery aims to restore the anatomical and functional integrity of the urinary tract, allowing affected women to regain their dignity and quality of life. However, in some cases, this surgical repair may fail. The objective of this study was to develop a predictive score to identify factors associated with the failure of surgical repair of obstetric urethro-vaginal fistula (FSROUVF) in the Democratic Republic of the Congo (DRC).

**Methods:**

This is an analytical cross-sectional study of 358 patients with obstetric urethro-vaginal fistula (OUVF) who received surgical repair. We conducted bivariate and multivariate analyses. Score discrimination was assessed using the receiver operating characteristic (ROC) curve, C-index, and score calibration according to the Hosmer-Lemeshow test.

**Results:**

Surgical repair of OUVF failed in 24.86% of cases (89/358). After logistic modelling, 6 criteria predicted FSROUVF: the use of intravaginal indigenous products (AOR = 3.59; 95% CI: 1.51–8.53), the presence of fibrosis (AOR = 6.37; 95% CI: 1.70–23.82), the presence of 2 or more fistulas in the same patient (AOR = 7.03; 95% CI: 3.14–15.72), the total urethral damage (AOR = 3.29; 95% CI: 1.36–7.95), the fistula size > 3 cm (AOR = 5.65; 95% CI: 2.12–15.01), and the postoperative infection (AOR = 351.10; 95% CI: 51.15–2409.81). A score of 0 to 14 was obtained, with a value ≤5 points indicating a low risk of FSROUVF, a value between 6 and 8 indicating a moderate risk, and a value ≥9 points corresponding to a high risk of FSROUVF. The area under the ROC curve of the score is 0.938 with a sensitivity of 60.67%, a specificity of 96.28%, a positive predictive value of 84.38%, and a negative predictive value of 88.10%.

**Conclusion:**

We report a FSROUVF rate in the DRC approaching a quarter of operative patients. Predictors of failure included fibrosis, presence of 2 or more fistulas, total urethral involvement, fistula size greater than 3 cm, postoperative infection, and use of intravaginal indigenous products. These factors are constitutive of the HEAL Africa score, which once validated, may have value in pre-operative counselling of patients. This study could be valuable for policy and strategies to address the problem of OUVF in the DRC and in resource limited settings more generally.

## Introduction

A urethro-vaginal fistula (UVF) is an abnormal communication between the urethra and the vagina. It results in uncontrolled loss of urine through the vagina. Compared to vesicovaginal fistulas (VVF) or recto-vaginal fistulas (RVF), UVF is a rare entity in the literature limited to clinical cases or a small series of patients [1]. Several authors do not separate UVF from VVF and consider urethral involvement as one of the clinical elements to be associated with VVF [2, 3]. According to Pushkar et al. [4], however, it is a conceptual error to consider UVF as synonymous with VVF. Notably, UVF is very different from VVF in terms of complications due to the risk of sphincter involvement. Etiologies of UVF in lower income countries differ from those found in higher income countries where they are usually a result of pelvic surgical procedures, such as stress incontinence surgery, urethral diverticulectomy, or anterior colporrhaphy [5, 6]. In lower income countries with limited access to emergency obstetric and neonatal care, UVF is mainly caused by obstetric trauma during childbirth [4, 7, 8].

UVF has a significant impact on women’s quality of life, leading to problems such as urinary incontinence, recurrent urinary tract infections, psychological disorders, and social stigma. Surgical repair aims to restore the anatomical and functional integrity of the urinary tract, allowing women to return to a normal life. Surgical repair is the primary mode of management to restore the anatomy and also function [9]. Many surgical techniques relating to this repair have been reported in the literature. Success rates for UVF repair vary across studies [4, 7]. In a series of 21 UVFs surgeries in Russia, Pushkar et al. [4] reported a surgical repair failure rate of 9.53%. However, the evaluation and comparison of surgical outcomes is challenged by the multiple injury combinations and lack of a standardized system of terminology, classification, data collection, and reporting. No previously published studies on UVF have assessed risk factors for failure of surgical repair because most involved clinical cases or only a small subset of patients.

Determining the rate of and identifying risk factors for failure of surgical repair of obstetric urethro-vaginal fistula (FSROUVF) can help improve the quality of patient care and overall fistula repair outcomes in the Democratic Republic of the Congo (DRC). The objective of this study was to determine the rate of FSROUVF and to identify its predictive factors. This will allow for the development of a predictive score adapted to our environment and will be useful in counselling patients pre-operatively.

## Materials and methods

### Study design, period, and population

This analytical cross-sectional study took place between January 2017 and December 2022 in general referral hospitals (GRH) in 7 provinces of the Democratic Republic of the Congo (DRC): HEAL Africa Hospital and GRH Beni in North Kivu province, GRH Wamba in Haut-Uélé province, GRH Lukonga in Kasai Central province, GRH Dr. Amu-Yasa-Bonga in Kwilu province, GRH Kipaka and GRH Kasongo in Maniema Province, GRH Karawa in North Ubangi Province, and GRH Katakokombe in Sankuru Province.

**Fig. 1:**
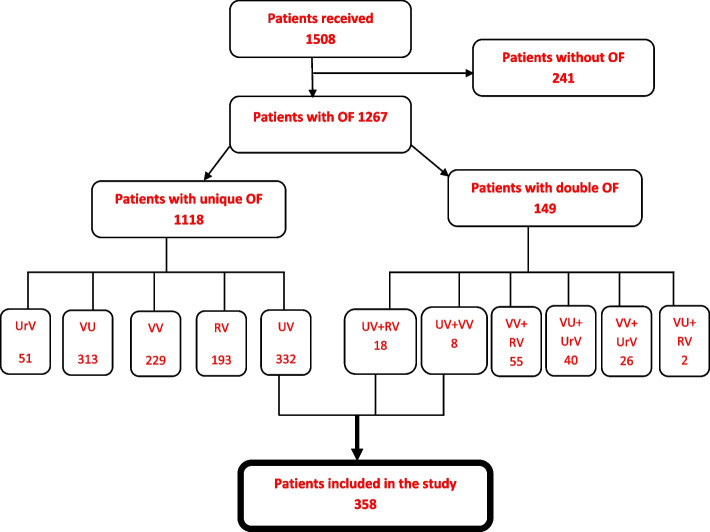
Flowchart of patients with Obstetric urethro-vaginal fistula. OF: Obstetric fistula, UrV: Uretero-vaginal, VU : Vesico-uterine, VV : Vesico-vaginal, RV : Recto-vaginal, UV : Uretro-vaginal

We recruited patients through 18 campaigns that offered free surgical operations for obstetric fistula, organized by the non-governmental organization HEAL Africa in collaboration with the National Ministry of Public Health of the DRC. These campaigns were designed to facilitate access to specialized care. During these campaigns, we registered 1508 patients who came in after awareness-raising, 1267 of whom had OF. Of these, 358 were confirmed to have OUVF and were included in the study (Fig. 1).

We included any woman presenting as an outpatient with obstetric UVF or referred for surgical repair during the study period. All patients were repaired by the same surgical team including an Obstetrician-Gynecologist and fistula surgeon, a general practitioner, a specialized urogynecological nurse, an operating room technician, and a nurse anesthetist.

At each surgical site, the team used the same antibiotic protocol comprised of a combination of ampicillin injection for 2 days, followed by oral amoxicillin and oral metronidazole for 5 days. This protocol proved highly effective in controlling infection during surgical repair campaigns.

After surgical repair, each patient benefited from strict, well-structured, post-operative follow-up for 14 consecutive days. During this period, 3 elements were particularly well monitored in each patient: drinking sufficient water, having continuous urine drainage via the bladder catheter, and sleeping in a dry bed. On day 14, we performed a methylene blue test to ensure the fistula was effectively closed. If there was doubt, bladder catheterization was extended for up to a week. Each patient was given a day of observation before discharge to make sure she was continent and could urinate independently. At the end of this evaluation, surgical repair was classified as either curative or a failure.

### Study variables

We collected sociodemographic characteristics including patient age at surgical repair (< 20 years, 20–29 years, 30–39 years, or ≥ 40 years), residence (rural or urban), educational level (none, primary or secondary), parity at surgical repair (1 or ≥ 2), and place of delivery at fistula onset (home or health facility). We also investigated the use of intravaginal indigenous products by patients as part of traditional fistula treatment that occurs in the community.

Regarding fistula clinical features, we collected the age of the fistula (< 1 year, 1–5 years, > 5 years), the number of fistulas in the same patient (1 or ≥ 2), fistula size (≤3 cm or > 3 cm), the association of UVF with another type of fistula in the same patient (yes or no), the presence of fibrosis (yes or no), the status of the urethra (partial or total involvement), and the number of previous surgical repair attempts (none or ≥ 1).

We also recorded the results of surgical repair (failure or success) as revealed by physical examination at the time of hospital discharge. Patients were divided into two groups based on the outcome of surgical repair which were:

- Failure of surgical repair defined as non-closure of the fistula. In these cases, the fistula was not fully closed, even though urine leakage often diminished considerably with or without continued micturition.
- Successful surgical repair defined as closure of the fistula. In these cases, the fistula was completely closed, with or without urinary incontinence. There was no leakage of urine at the fistula site.

### Operational definitions

Perioperative hemorrhage was defined as any bleeding > 300 ml from the obstetric fistula operative site during the surgical procedure.

Post-operative infection referred to any infection occurring at the surgical site after obstetric fistula repair surgery. This was a clinical diagnosis noted by erythema, swelling, and warmth at the surgical site, in addition to persistent or increased pain after surgery, and/or purulent or abnormal discharge from the surgical site.

Fistula size was determined by measuring the largest diameter of the fistula with a metal probe. After taking the length of the largest diameter of the fistula between the tip of the metal probe and the tips of the right thumb and forefinger holding the probe, this measurement was plotted on a graduated bar. 

Fibrosis or fibrotic scarring was assessed in terms of the presence of scar tissue around the fistula. Fibrosis was either present or absent and this was assessed clinically by the surgeon.

Urethral involvement was determined by the surgeon and categorized as partial when part of the urethra was still identifiable and mobilized, or as total involvement when the urethra was completely absent.

Indigenous or traditional products included any products made from herbs that had been used vaginally by patients to treat fistula prior to surgical repair.

### Statistical analysis

Statistical analyses were carried out using STATA 16 software. Characteristics of patients with FSROUVF were compared with those who had successful surgical repair. Analyses examined the associations between each of the patient demographic variables and fistula characteristics (independent variables) with FSROUVF (dependent variable). The association between an explanatory variable and FSROUVF was measured by calculating odds ratios (OR) and their 95% confidence intervals (95% CI). Pearson’s Chi-square test was used to compare the observed proportions. Statistical significance was set at *p* < 0.05.

All variables with significance less than 0.2 in the unifactorial analysis were included in a multivariate logistic regression analysis.

For the construction of the multivariate logistic model, the stepwise selection method was chosen at the threshold of *p* < 0.05. The logistic model thus made it possible to analyze the contribution of each explanatory variable to FSROUVF in the presence of the other independent variables.

Score discrimination was used to determine how well the HEAL Africa score separated patients with and without FSROUVF [10]. The discrimination of the logistic model was assessed by calculating the area under the ROC (receiving operating characteristic) curve, which plotted the sensitivity as a function of the specificity complement (1– specificity). The calibration of the score was done by the Hosmer-Lemeshow test. The sensitivity, specificity, and percentage of cases correctly classified were determined using the C-index. Evaluation of model robustness was done by the bootstrap method.

A predictive risk score was derived at the end of the statistical analysis. To develop a screening tool to predict FSROUVF, points were assigned to each risk factor selected in the logistic model. To make it simple and usable, the score was estimated using the rounded values of these coefficients [11]. The risk probabilities of the FSROUVF based on the constructed score values were also calculated.

## Results

A total of 358 patients with OUVF underwent surgical repair (Fig. 1). The repair failed in 24.86% of cases (89/358).

Table 1 shows that there was no statistically significant association between FSROUVF and sociodemographic characteristics such as patient age at time of repair, parity at time of repair, residence, education, and place of delivery (*p* > 0.05). In contrast, a statistically significant association was found between FSROUVF and intravaginal indigenous products use (crude OR = 4.64 [2.65–8.14]; *p* < 0.0001).

**Table 1:** Sociodemographic characteristics and history in correlation with failure of surgical repair in patients with urethro-vaginal obstetric fistula in the DRC (*N* = 358)

Variable	Result of surgical repair of obstetric urethro-vaginal fistula				Total (*N* = 358)	Crude OR [95% CI]	*P*-value
	Failed (*n* = 89)		Success (*n* = 269)				
**Age at repair**							
< 20 years	8	17.78%	37	82.22%	45	1.00	
20–29 years	40	28.57%	100	71.43%	140	1.85 [0.79–4.32]	0.2144
30–39 years	22	23.66%	71	76.34%	93	1.43 [0.58–3.53]	0.5723
≥ 40 years	19	23.75%	61	76.25%	80	1.44 [0.57–3.62]	0.5806
**Residence**							
Rural	74	26.15%	209	73.85%	283	1.42 [0.76–2.65]	0.3446
Urban	15	20.00%	60	80.00%	75	1.00	
**Level of Education**							
None	31	26.50%	86	73.50%	117	1.00	
Primary	44	28.21%	112	71.79%	156	1.09 [0.64–1.87]	0.8602
Secondary	14	16.5%	71	83.5%	85	0.55 [0.27–1.11]	0.1287
**Place of delivery**							
Home	36	28.13%	92	71.88%	128	1.31 [0.80–2.14]	0.3479
Health facility	53	23.04%	177	76.96%	230	1.00	
**Parity at time of repair**							
1	56	23.63%	181	76.37%	237	1.21 [0.73–2.00]	0.5317
≥ 2	33	27.3%	88	72.7%	121	1.00	
**Pre-operative use of intravaginal indigenous products**							
No	19	11.24%	150	88.76%	169	1.00	
**Yes**	**70**	**37.04%**	**119**	**62.96%**	**189**	**4.64 [2.65–8.14]**	**< 0.0001**

In terms of clinical features, Table 2 shows that fistula age and a previous fistula repair attempt were not significantly associated with FSROUVF (*p* > 0.05). However, a significant association was noted between FSROUVF and the number of fistulas in the same patient, presence of OUVF with another type of fistula, status of the urethra, fibrosis, fistula size, and postoperative complications. FSROUVF was greater in the presence of two or more fistulas in the same patient (crude OR = 10.92 [6.05–19.71]; *p* < 0.0001), in the presence of another type of fistula associated with UVF (crude OR = 4.75 [2.09–10.79]; *p* < 0.0002), when the urethra was fully damaged (crude OR = 5.67 [3.29–9.76]; *p* < 0.0001), in the presence of fibrosis (crude OR = 8.42 [4.28–16.53]; *p* < 0.0001), when the fistula was larger than 3 cm (crude OR = 9.07 [4.99–16.49]; *p* < 0.0001), in the presence of perioperative hemorrhage (crude OR = 4.90 [2.26–10.66]; *p* < 0.0001) and in the presence of postoperative infection (crude OR = 27.77 [8.97–115.19]; *p* < 0.0001).

**Table 2:** Clinical features correlated with failure of surgical repair in patients with urethro-vaginal obstetric fistula in the DRC (*N* = 358)

Variable	Result of surgical repair of obstetric urethro-vaginal fistula				Total (*N* = 358)	Crude OR [95% CI]	*P*-value
	Failed (*n* = 89)		Success (*n* = 269)				
**Age of fistula**							
< 1 years	18	23.08%	60	76.92%	78	1.00	
1–5 years	26	25.74%	75	74.26%	101	1.16 [0.58–2.30]	0.8137
> 5 years	45	25.14%	134	74.86%	179	1.12 [0.60–2.09]	0.8448
**Previous fistula repair attempt**							
No	61	28.37%	154	71.63%	215	1.00	
≥ 1	28	19.58%	115	80.42%	143	0.61 [0.37–1.02]	0.0784
**Number of fistulas in the same patient**							
1	43	14.93%	245	85.07%	288	1.00	
**≥ 2**	**46**	**65.71%**	**24**	**34.29%**	**70**	**10.92 [6.05–19.71]**	**< 0.0001**
**Association with other types of fistula**							
No	74	22.29%	258	77.71%	332	1.00	
**Yes**	**15**	**57.69%**	**11**	**42.31%**	**26**	**4.75 [2.09–10.79]**	**0.0002**
**Status of the urethra**							
Partial damage	22	11.17%	175	88.83%	197	1.00	
**Total damage**	**67**	**41.61%**	**94**	**58.39%**	**161**	**5.67 [3.29–9.76]**	**< 0.0001**
**Presence of fibrosis**							
No	11	7.01%	146	92.99%	157	1.00	
**Yes**	**78**	**38.81%**	**123**	**61.19%**	**201**	**8.42 [4.28–16.53]**	**< 0.0001**
**Fistula size**							
≤ 3 cm	16	8.21%	179	91.79%	195	1.00	
**> 3 cm**	**73**	**44.79%**	**90**	**55.21%**	**163**	**9.07 [4.99–16.49]**	**< 0.0001**
**Complications**							
No	51	16.94%	250	83.06%	301	1.00	
**Peri-operative hemorrhage**	**15**	**50.00%**	**15**	**50.00%**	**30**	**4.90 [2.26–10.66]**	**< 0.0001**
**Post-operative infection**	**23**	**85.19%**	**4**	**14.81%**	**27**	**27.77 [8.97–115.19]**	**< 0.0001**

After logistic regression, presence of fibrosis, patient having 2 or more fistulas, total urethral damage, fistula size > 3 cm, use of intravaginal indigenous products, and post-operative infection were all associated with FSROUVF, as illustrated in Table 3.

**Table 3:** Coefficients and scores of logistic regression model of risk of FSROUVF

Variable	Adjusted OR [95% CI]	Coefficient	*p*-value	Score
Fibrosis	6.37 [1.70–23.82]	1.85	0.0059	2
Number of fistulas ≥2	7.03 [3.14–15.72]	1.95	< 0.0001	2
Fistula size > 3 cm	5.65 [2.12–15.01]	1.73	0.0005	2
Total urethral damage	3.29 [1.36–7.95]	1.19	0.0082	1
Pre-operative use of intravaginal indigenous products	3.59 [1.51–8.53]	1.28	0.0039	1
Post-operative infection	351.10 [51.15–2409.81]	5.86	< 0.0001	6

**Fig. 2:**
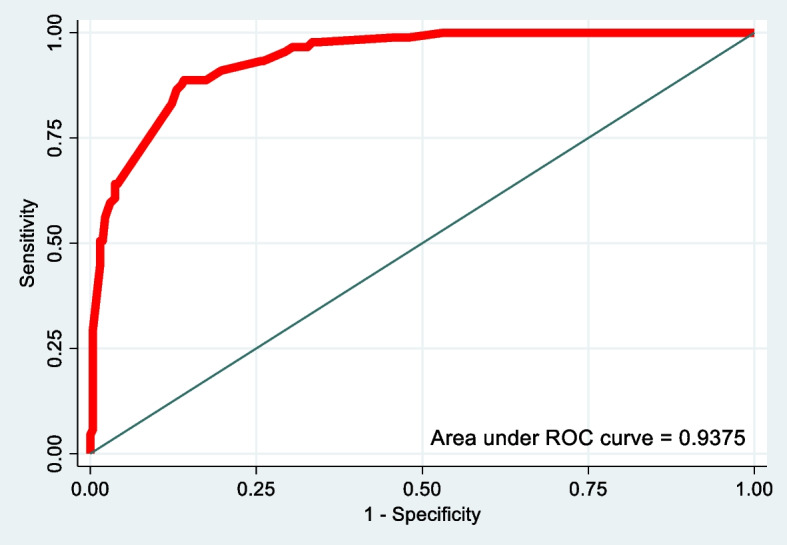
ROC curve of the HEAL Africa score in the prediction of FSROUVF

The HEAL Africa score was constructed from the logistic regression model. Each risk factor was weighted by a regression coefficient representing the weight of the variable in the score calculation, with all obtained scores illustrated below (Table 3). The area under the ROC curve for the HEAL Africa score was 0.938 (Fig. 2), showing clear discrimination between patients who will have FSROUVF and those who will not.

Each of the 6 criteria corresponds to a given number of points, with a total possible score of 14 points. Higher scores indicate higher risk of FSROUVF. The risk probabilities of FSROUVF based on constructed score values were calculated and are presented in Table 4. A score ≤ 5 points defines a group of patients at low risk of FSROUVF; a score between 6 and 8 points defines a moderate risk of FSROUVF and a score ≥ 9 points presents a high risk of FSROUVF.

**Table 4:** Probability of FSROUVF by score according to logistic regression model

Score obtained	Probability of failure*
0	0.19%
1	0.48%
2	1.21%
3	3.03%
4	7.38%
5	16.90%
6	34.19%
7	50.01%
8	77.21%
9	89.64%
10	95.67%
11	98.26%
12	99.31%
13	99.73%
14	99.89%

*obtained from the formula: *p* = 1/1 + exp. (−6.28–0.938 x score)

This HEAL Africa score had a sensitivity of 60.67% and a specificity of 96.28%. The positive predictive value was 84.38% and the negative predictive value was 88.10%.

## Discussion

This study determined the rate of FSROUVF in the DRC to be 24.86% (89/358). FSROUVF rates are known to vary depending on the cause, the complexity of the fistula, and the expertise of the surgeon. Previous studies [7, 12–14] have reported case series of urethro-vaginal fistula repair with failure rates ranging from 8 to 30%. The success of repair can be influenced by the surgical technique used. Martius’ procedure, which consists of interposition of a flap of labial fat between the fistula and vagina, has been successfully used to repair UVF and is noted to have improved success rates [15, 16]. In UVF surgery, all efforts to restore the patient’s anatomy should prioritize achieving a functionally satisfactory result concerning the urethral sphincter mechanism [7].

This multifactorial analysis modelled the risk factors associated with FSROUVF in the DRC, revealing FSROUVF predictors which were generally consistent with those reported in earlier studies [17–19]. These included the presence of fibrosis, the patient having 2 or more fistulas, total urethral damage, fistula size > 3 cm, and postoperative infection. Our current work added the use of intravaginal indigenous products used by traditional healers to treat urogenital fistulas.

Fibrosis, multiple fistulas in the same patient, total urethral involvement, and fistula size > 3 cm compromise the success of surgical fistula repair by restricting the mobility of surrounding tissues, which can lead to excessive strain on the suture and subsequent dehiscence of the repair [17, 18, 20]. This increases the complexity of the surgical procedure, with additional difficulties related to dissection, closure of fistulas, and reconstruction of surrounding tissue. Subsequently, the local vascularization is impaired, thus compromising normal healing and consolidation of sutures [20]. Notably, the pre-operative use of intravaginal indigenous products leads to hardening of the vaginal walls and accentuates the development of fibrosis around the fistula which can complicate surgical repair by making the tissues less flexible and more difficult to suture. Use of intravaginal indigenous products is a phenomenon often observed in African women living in rural areas. In many African communities, the use of indigenous intravaginal products is rooted in traditional practices. These products are made from herbs often used for feminine care purposes or in preparation for events such as childbirth and, as in our study, the treatment of obstetric fistula. However, when used prior to obstetric fistula repair surgery, these products can have adverse effects. Hardening of the vaginal walls and increased fibrosis formation complicate the repair. As the tissue becomes less flexible, it may be more difficult to suture the fistula effectively, compromising the success of the operation. This underscores the importance of comprehensive pre-operative care, patient education and adherence to medical guidelines to ensure the best possible results for obstetric fistula repairs. As with any other fistula repair, strict adherence to surgical principles is important for successful reconstructive surgery, including careful re-approximation of mucosal edges, multilayer closure, and tension-free repair, as well as maintaining a hemostatic surgical field throughout the procedure [15, 21]. For complicated cases of UVFs, such as those occurring in fibrosed or irradiated tissue, recurrent fistulas, or larger fistulas where tension-free closure cannot be achieved, a fat pad interposition graft may be used [15].

The present study found that OUVF repair in a patient with postoperative infection was 351 times more likely to result in FSROUVF (AOR = 351.10; 95% CI: 51.15–2409.81). As Aynie et al. had also reported [22], it is evident that post-operative infection is critical because it has significant negative implications. Infection can lead to deterioration in tissue quality, making suturing difficult or impossible. In addition, the inflammation associated with the infection can compromise local blood circulation, leading to poor tissue vascularization and a decreased supply of nutrients needed for healing. Thus, the importance of post-operative management for successful fistula repair needs to be emphasized and has been noted by other authors [4]. For instance, the prevention of urinary tract infections before repair and during the post-operative period is key in avoiding complications related to fistula surgery and promoting healing.

Our analysis allowed for the development of a tool, the HEAL Africa score, to identify the risk of FSROUVF. In contrast to patient characteristics, our results highlight the negative influence of fistula characteristics on repair outcomes. FSROUVF was significantly associated with fibrosis, patient having 2 or more fistulas, total urethral damage, fistula size greater than 3 cm, postoperative infection, and use of intravaginal indigenous products. The ROC curve helped to define a threshold that was both sensitive and specific enough to screen patients at risk of developing FSROUVF. This threshold remains limited to a sensitivity of 60.67% and a specificity of 96.28%.

Results are based on data collected from patients in several provinces of the DRC. To our knowledge, this study is the first to correlate risk factors with fistula surgical repair outcomes in our context and also the first to develop a FSROUVF predictive score. This simple tool will inform clinicians in our environment of the likelihood of identifying patients at risk of surgical fistula repair failure prior to each procedure and will guide surgical decision-making and patient counselling. Since the first surgical repair attempt always has the best chance of success, all risk factors must be considered to improve outcomes during fistula repair surgery.

OUVF should be managed by a multidisciplinary team including an Obstetrician-Gynecologist consultant, a urogynecologist, a surgeon, and an anesthetist. Healthcare providers should recognize patients at risk of FSROUVF and transfer them to a tertiary referral hospital for appropriate management. To avoid recurrence, cesarean delivery should be planned for any future pregnancies [23].

The strengths of the present study include the relatively large sample size for a poorly documented anatomoclinical entity such as UVF and the suitability of including all study variables for each patient treated for UVF. However, among the weaknesses of this study, it is important to acknowledge the challenges associated with thoroughly assessing patients with continued incontinence despite having a closed fistula. For these types of patients, a urodynamic study would typically be conducted to gain a comprehensive understanding of their condition. It is possible that some incontinent patients with closed fistula at the time of discharge from the hospital may have become continent in the following weeks or months. Thus, it is important to conduct long-term follow-up assessments to accurately classify the patients. Although the present study did not look at urinary incontinence in detail, this will be included in future research. Another limitation of this study was the inability to access some factors, for example, body mass index, smoking history, and diabetes mellitus, which have reportedly been associated with OUVF treatment results. These variables should be considered in future studies.

## Conclusion

The current study in the DRC reports a FSROUVF rate approaching a quarter of all treated patients. We identified FSROUVF predictors as the presence of fibrosis, patient having 2 or more fistulas, total urethral damage, fistula size greater than 3 cm, the use of intravaginal indigenous products, and postoperative infection. Collectively, these factors constitute the HEAL Africa score which may allow surgeons to develop individualized treatment plans and implement appropriate strategies to minimize the risk of FSROUVF. This study could be valuable for policy and strategies to address the problem of OUVF in the DRC, and in resource limited settings more generally. Improved surgical techniques and interdisciplinary approaches can also help improve surgical repair outcomes for OUVF, thereby improving the quality of life of patients with this debilitating condition.

## Data Availability

The datasets used and/or analyzed during the current study are available from the corresponding author on reasonable request.

## Abbreviations

AOR: Adjusted odds ratio
DRC: Democratic Republic of the Congo
FSROUVF: Failure of surgical repair of obstetric urethro-vaginal fistula
GRH: General referral hospital
OF: Obstetric fistula
OR: Odds ratio
OUVF: Obstetric urethro-vaginal fistula
ROC: Receiving operating characteristic
UVF: Urethro-vaginal fistula
VV: Vesico-vaginal fistula
95% CI: 95% Confidence intervals
